# RAF Kinase Inhibitory Protein Expression and Phosphorylation Profiles in Oral Cancers

**Published:** 2016-09-01

**Authors:** DP Hallums, R Gomez, AP Doyle, CT Viet, BL Schmidt, NA Jeske

**Affiliations:** 1 Departments of Oral and Maxillofacial Surgery, University of Texas Health Science Center at San Antonio, USA; 2 Departments of Pharmacology, University of Texas Health Science Center at San Antonio, USA; 3 Departments of Physiology, University of Texas Health Science Center at San Antonio, USA; 4 Department of Oral Maxillofacial Surgery, New York University, USA; 5 Department of Oral Maxillofacial Surgery, Bluestone Center for Clinical Research, New York University, USA

**Keywords:** Oral cancers, Raf kinase, Phosphorylation

## Abstract

Raf Kinase Inhibitory Protein (RKIP) expression has been profiled for a number of unique tissue cancers. However, certain tissues have not been explored, and oral and oropharyngeal cancers stand out as high priority targets, given their relatively high incidence, high morbidity rate, and in many cases, preventable nature. The purpose of this study was to examine changes in RKIP expression and phosphorylation in tissues resected from oral cancer patients, and compare to results generated from immortalized cell lines raised from primary oral cancer tissues, including oral squamous cell carcinoma line 4 (SCC4) and human squamous cell carcinoma line 3 (HSC3). Out of 4 human samples collected from male and female patients across various ages with variable risk factors, we observed an across the board reduction in RKIP expression. Two human samples demonstrated a significant increase in phosphorylated RKIP when normalized to total RKIP, however all 4 were increased when normalized to total cellular protein. The immortalized oral cancer cell culture HSC3 revealed significant increases in phosphorylated RKIP with no change in total RKIP expression, while line SCC4 demonstrated an increase in both total and phosphorylated RKIP. Results presented here indicate that oral cancers behave similarly to other cancers in terms of changes in RKIP expression and phosphorylation, although immortalized cell line expression profiles significantly differ from human tissue biopsies.

## Introduction

Oral squamous cell carcinoma is the most common cancer in the orofacial cavity, accounting for approximately 90% of all cancers of the mouth [1]. Te American cancer society estimates that approximately 37,000 people will be diagnosed with oral or oropharyngeal cancer in 2014, and an estimated 7,300 people will die. These values represent 2.22% of the American population, which is greater than many other cancer types. However, little proteomic profiling has been conducted on oral cancers, leaving many clinical researchers with few potential targets for therapeutic treatment. In this report, we tracked the expression profile for Raf Kinase Inhibitory Protein (RKIP, also known as phosphatidylethanolamine-binding protein or PEBP) in oral cancer cells.

RKIP is a scaffolding protein capable of binding to and inhibiting Raf kinase [2]. Raf kinase classically participates in the Ras/Raf/MEK/ERK kinase cascade that transfers mitogenic signals from the cell membrane to the nucleus [3]. Therefore, RKIP has the capacity to interrupt cell differentiation and growth, depending on the cell signal being received. In its monomeric form, RKIP binds to Raf-1 kinase, preventing downstream signal transduction, thereby maintaining transcriptional events at basal levels. However, upon cell stimulation, phosphorylation of RKIP at Ser153 results in dimerization of the scaffolding protein, causing it to switch molecular attractions from Raf-1 to G-protein Receptor Kinase 2 (GRK-2) [4]. This switch can allow Raf-1 to signal downstream in an unrestricted fashion, resulting in cellular transcription, differentiation, and growth. Additional RKIP scaffolding to NFκB, which normally mediates cellular apoptosis, can also be interrupted by phosphorylation, resulting in anti-apoptotic signaling mechanisms that preserve and prolong cellular integrity [5]. Te participation of RKIP in these ubiquitous signaling pathways highlights the importance of this protein to cancer formation and metastasis.

Previous studies identify RKIP as an important protein determinant in many types of cancers, including prostate, melanoma, colorectal, liver, and breast [6-10]. Indeed, studies report significant down-regulation of RKIP expression in differentiated gastric cancer cells [11] and a number of solid tumors including prostate [7], breast [12], colorectal [13], and melanoma [14]. Given its role in multiple signaling events that control significant factors of cell survival, down-regulated RKIP expression would have dramatic results on cellular growth and phenotype. Further, RKIP phosphorylation, caused by PKC activation, is associated with poor outcomes in certain cancers including colon [15]. Therefore, we sought to identify RKIP phosphorylation and expression profiles in tissue biopsies resected from oral cancer patients, and compare results to those from widely used oral cancer cell lines derived from squamous cell carcinomas.

## Materials and Methods

### Cell/Animal/Patient samples

Oral squamous cell carcinoma line 4 (SCC4) and human squamous cell carcinoma line 3 (HSC3) cells were grown as previously described [16]. Cell cultures were left untreated, and in the presence of 2% serum for 24 hours prior to harvesting for protein extraction.

All procedures utilizing animals were approved by the Institutional Animal Care and Use Committee of the University of Texas Health Science Center at San Antonio and were conducted in accordance with policies for the ethical treatment of animals established by the National Institute for Health. Male Sprague-Dawley rats 175 – 200g in weight (Charles River Laboratories, Wilmington, MA) was used for trigeminal ganglia (TG) dissection, as described previously [17].

The study was approved by the Institutional Review Board of New York University College of Dentistry and University of California San Francisco (UCSF). All patients provided written informed consent in accordance with the Declaration of Helsinki. Patients were enrolled with the following inclusion criteria: 1) biopsy-proven HNSCC, and 2) no history of prior surgical, chemotherapeutic, or radiation treatment for oral SCC. Demographic information was collected for each patient including age, sex, ethnicity, oral SCC location (tongue, floor of mouth, buccal mucosa, gingival, palate), tumor size (greatest dimension based on clinical examination), and evidence of metastasis. At the time of surgical resection a 5×5 mm piece of oral cancer was collected. A normal piece of oral mucosa measuring 5×5 mm was collected from a contralateral, anatomically matched site.

### Western blotting

Cells and tissues were homogenized by 20 strokes in a Potter-Elvejm homogenizer on ice in homogenization buffer (25mM HEPES, 25mM sucrose, 1.5mM MgCl_2_, 50mM NaCl, pH to 7.2) with protease and peptidase inhibitors (aprotinin10μg/ml, leupeptin 10μM, pepstatin 1μg/ml, phenylmethylsulfonyl fluoride (PMSF, 10μg/ml), sodium orthovanadate 100μg/ml) added immediately prior to harvesting. Homogenates were incubated on ice 15 min, then lysed with 1% Triton X-100 via 20 passes through a 25g tuberculin needle, and incubated on ice 10 min. Samples were centrifuged at 1000g for 1 min at RT to precipitate un-lysed cells, and protein content was determined by Bradford analysis [Bradford, 1976 #301]. 25 μg aliquots were resolved by sodium dodecyl sulfate-polyacrylamide gel electrophoresis (SDS-PAGE) and transferred to polyvinyl difluoride (PVDF, EMD Millipore, Billerica, MA) membranes for Western blotting, using antibodies specific to phosphorylated RKIP (Ser 153, SantaCruz Biotechnology, Santa Cruz, CA), total RKIP (SantaCruz Biotechnology) and β-actin (SantaCruz Biotechnology). Appropriate secondary antibodies (GE Healthcare Life Sciences, Piscataway, NJ) were utilized with enhanced chemiluminescence (GE Healthcare Life Sciences) to visualize protein immunoreactivity. Samples were independently analyzed 3-4 times; images are representative of all trials. Pixel densities of scanned Western blots were quantified using NIH image 1.62, with samples normalized as indicated in figures.

## Results

### RKIP expression profile in oral cancer cell lines

**Figure 1:**
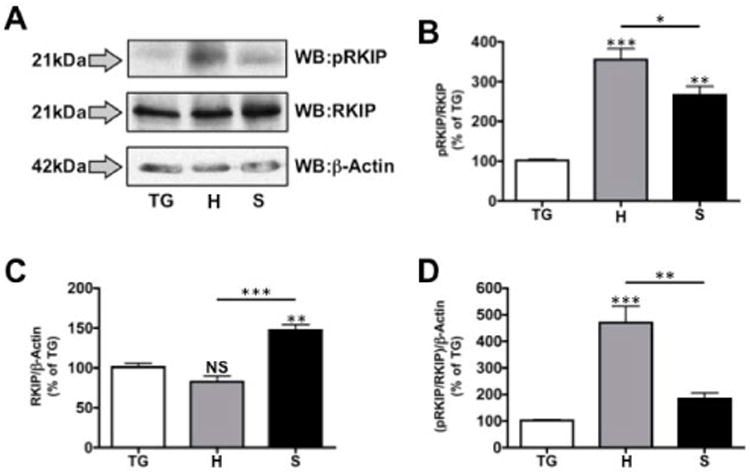
RKIP phosphorylation and expression in oral cancer cell lines Primary trigeminal neuron cultures (TG), HSC3 (H) and SCC4 (S) cell lines were lysed and prepared for proteomic analysis by Western blot. **A.** Proteins were identified using antibodies specific for phospho Ser153 RKIP (pRKIP), total RKIP (RKIP) and β-Actin. Protein molecular weights are shown to the left of the lots. Densitometry was performed on immunoreactive bands, with pRKIP normalized to total RKIP (**B**), total RKIP normalized to β-Actin (**C**) and pRKIP normalized to RKIP/β-Actin (**D**). Results shown are representative of 3 independent trials; statistics determined by one-way ANOVA with Bonferroni post-hoc correction.

Oral cancers are widely studied for their invasive morbidity, yet little has been reported on expression profiles of transcriptional regulators. RKIP, an important modulator of the Ras/Raf/MEK/ERK kinase cascade, has been characterized in multiple cancer types, except for oral. To begin our investigation, we probed for phosphorylated and total RKIP expression in immortalized oral cancer cells lines oral squamous cell carcinoma line 4 (SCC4) and human squamous cell carcinoma line 3 (HSC3). Cell lysates were generated of these cells under naïve, low serum conditions, to more closely represent physiological signifcance. Furthermore, we compared the protein expression profiles of these cancer cell lines to that of a primary cell model, trigeminal ganglia (TG) neurons. The inclusion of other immortalized cell lines for control purposes would have confounded result interpretation given that most cell lines are derived from cancer tissues, and that any immortalized cell line would represent an abnormal transcriptional environment. In (Figure 1), Western blot analysis was performed on protein samples taken from cell lysates of cultured TG neurons, HSC3 cells, and SCC4 cells. Blots were probed for phosphorylated RKIP (pRKIP, Ser 153), total RKIP, and β-actin (loading control), and analyzed for densitometry to quantify expression between cell lines. Firstly, we observed a significant increase in phosphorylated RKIP when normalized to total RKIP in both cell lines (Figure 1A-B). Additionally, we observed a 50% increase in total RKIP in SCC4 cells over the TG control, while HSC3 cells reveal little change in expression (Figure 1A and 1C). We then conducted further normalization to account for changes in RKIP expression (normalized to β-actin) when measuring the significance of RKIP phosphorylation in the HSC3 and SCC4 cell lines. As demonstrated in (Figure 1D), HSC3 cells displayed a 5–fold increase in phosphorylated RKIP when compared to our control TG cells, while SCC4 cells displayed less than a 1-fold increase. Together, these data indicate differential RKIP phosphorylation and expression profiles between commonly used oral cancer cell lines.

### RKIP expression profile in oral cancer tissue biopsies

**Figure 2:**
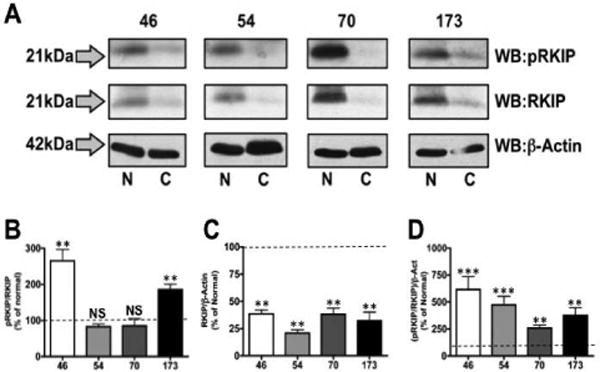
RKIP phosphorylation and expression in oral cancer tissue biopsies Tissue biopsies from patient oral cancers (46, 54, 70, and 173) and matched non-cancerous controls were homogenized, lysed and prepared for proteomic analysis by Western blot. **A.** Proteins were identified using antibodies specific for phosphorylated Ser153 RKIP (pRKIP), total RKIP (RKIP) and β-Actin. Protein molecular weights are shown to the left of the lots. Densitometry was performed on immunoreactive bands, with pRKIP normalized to total RKIP (**B**), total RKIP normalized to β-Actin (**C**) and pRKIP normalized to RKIP/β-Actin (**D**). Dashed line denotes control expression, set at 100%. Results shown are representative of 4 independent trials; statistics determined by one-way ANOVA with Bonferroni post-hoc correction.

We next performed similar analysis on tissue biopsies of oral cancers collected from patients. In this analysis, we compared changes in RKIP expression and phosphorylation between cancerous biopsies and healthy tissue control samples taken from the same patient. Therefore, for each patient, normal tissue (N) was compared to cancerous tissue (C). In (Figure 2), Western blot analysis was performed on lysates generated from tissue homogenization, and probed for in a similar fashion as in Figure 1. Importantly, we observed differential RKIP phosphorylation profiles between patient biopsies, demonstrating significant increases (approximately 250% of control tissue (sample 46) and 200% (sample 173), (Figure 2A and 2B) in two patients. Furthermore, all samples produced significantly reduced total RKIP expression profiles (Figure 2A and 2C). As in Figure 1, we normalized phosphorylated RKIP measurements to those of RKIP normalized to β-actin, to accurately identify changes in modified RKIP while accounting for changes in total RKIP expression. In (Figure 2D), we observed a significant increase in pRKIP/RKIP/β-actin for all patient samples over control tissues, indicating that despite reductions in total RKIP expression, the phosphorylated state of the protein is significantly higher.

Patient data were examined to determine whether patient history and/or tumor characteristics could account for sample-to-sample differences observed in (Figure 2). (Table 1) outlines de-identified information on each of the patients that cancerous and contralateral normal tissue samples were taken from. These data sets include, age, sex, tumor/node/metastasis (TNM) stage, tumor site, and patient tobacco history. While the buccal mucosa SCC cancer biopsy (P46) provided the highest pRKIP/RKIP/β-Actin expression profile, there was little else in the way of correlative descriptive patient data that could explain the remaining differential RKIP expression profiles. Taken together, these data support a strong case for reduced RKIP expression and increased phosphorylated RKIP in human cancer tissue biopsies. Collectively, these data identify an important expression profile that is mirrored by many other cancers.

## Discussion

RKIP transcript and protein expression has been characterized in numerous cancer types, except for oral cancers. Since oral squamous cell transformation rate is relatively high compared to other cancers, changes to mitogenic signaling modulators such as RKIP are highly significant to understanding the pathogenesis of the disease. Here, we performed proteomic analysis of RKIP phosphorylation and expression from two well-studied immortalized oral cancer cell lines and multiple squamous cell carcinoma biopsies. Our results indicate that RKIP expression is reduced in cancerous tissue biopsies, similar to what is found in other cancer types [7,11-14]. Furthermore, we observed increased RKIP phosphorylation in tissue biopsies, which parallel observations in other cancer models, and is deemed a determinant of poor chemotherapeutic prognosis [15]. Interestingly, only one of the immortalized cell lines (HSC3) provided similar metrics of RKIP expression and phosphorylation, despite both in vitro cell lines being used commonly as oral cancer models. Importantly, HSC3 cells provide a unique in vitro model that mirrors the RKIP phosphorylation and expression profile characterized in human oral cancer tissues.

Te role of RKIP in cancer and tumor progression was first identified in prostate cancer cells in 2003 [7]. Here, investigators identified low RKIP expression in LNCaP prostate cancer cell lines, and were able to reduce in vitro invasive ability by over-expressing RKIP. In vivo, increased RKIP expression in implanted C4-2B cells reduced spontaneous metastasis, but did not affect primary tumor growth rate, initially identifying RKIP as a metastasis tumor suppressor gene. This conclusion has been reproduced in several other cancer types, including breast and urinary bladder [18-20]. Therefore, RKIP expression may serve as an important prognosticator of metastatic potential of oral cancers, and can be easily determined by proteomic means as demonstrated here. Indeed, proteomic analyses are used currently to detect cell surface markers in breast cancer [21], whole cell proteomes in prostate cancer [22], and transcription factor expression profiles for melanomas [23]. Taken together, differential RKIP characterization of cancerous oral tissues could provide unique insight into their metastatic potential.

RKIP phosphorylation primarily serves to redirect the protein away from its inhibition of Raf Kinase and into a scaffolding role with other signaling proteins, such as G-protein receptor kinase 2 [4,24]. It is interesting to note that we observed a significant increase in RKIP phosphorylation in all cell and tissue models, although normalized significance was only present in the HSC3 cell line and in each of the tissue biopsies. Increased RKIP phosphorylation would allow for increased Raf signaling downstream to multiple mitogenic transcription factors, which can stimulate cell growth and division, and also suppress normal RKIP expression through activation of a Snail zinc-finger transcription repressor [25]. Therefore, one could argue that phosphorylation of RKIP by PKC-signaling transduction contributes to the initial step towards tissue metastasis by effectively reducing overall RKIP expression via transcriptional repression.

From results presented here, proteomic analysis of oral cancers could serve as an important screening tool to predict metastatic potential and assist with determining optimal treatment for the patient.

## Figures and Tables

**Table 1:** Oral Cancer Patient and Tumor Diagnoses Vital records were kept for the oral cancer patients (P46, P54, P70, and P173), including age, sex, tumor/nodal/metastasis (TNM) stage, tumor site, and tobacco history.

	P46	P54	P70	P173
pRKIP/RKIP	265.6	82.7	85.1	185.3
RKIP/β-Actin	38.6	20.8	38.3	22.3
pRKIP/RKIP/β-Actin	615.7	474.2	260.1	375.2
Age	59	56	60	77
Sex	M	M	W	M
TNM Stage	T_2_N_1_M_0_	T_1_N_0_M_0_	T_3_N_2B_M_0_	T_3_N_2B_M_0_
Tumor Site	Buccal Mucosa SSC	Tongue SSC	Mandibular Gingiva SSC	Mandibular Gingiva SSC
Tobacco History	None	30 pack/yr	None	5 pack/yr
